# Esketamine nasal spray versus quetiapine XR in adults with treatment-resistant depression: a secondary analysis of the ESCAPE-TRD randomized clinical trial

**DOI:** 10.1017/S1092852924002451

**Published:** 2025-01-17

**Authors:** Roger S. McIntyre, Gregory Mattingly, Yordan Godinov, Jozefine Buyze, Ibrahim Turkoz, Patricia Cabrera, Manish Patel, Larry Martinez, Mai Himedan, Oliver Lopena

**Affiliations:** 1 Department of Psychiatry, University of Toronto, Toronto, ON, Canada; 2 Midwest Research Group, St. Charles, MO, USA; 3 Janssen EMEA, Sofia, Bulgaria; 4 Janssen Pharmaceutica NV, Beerse, Belgium; 5 Janssen Research and Development, LLC, a Johnson & Johnson company, Titusville, NJ, USA; 6 Janssen Scientific Affairs, LLC, a Johnson & Johnson company, Titusville, NJ, USA

**Keywords:** Esketamine, major depressive disorder, quetiapine, remission, treatment-resistant depression

## Abstract

**Objective:**

Esketamine nasal spray (ESK) is approved in combination with an oral antidepressant (OAD) for the treatment of adults with treatment-resistant depression (TRD); however, direct comparisons with atypical antipsychotics for TRD are limited. This secondary analysis of the ESCAPE-TRD study compared rates of remission and response, and improvements in depressive symptoms over time, between ESK and quetiapine extended-release (XR) in patients with TRD treated in accordance with US prescribing information (USPI).

**Methods:**

ESCAPE-TRD (NCT04338321) was a randomized, open-label, rater-blinded phase 3b trial investigating ESK versus quetiapine XR for acute and maintenance treatment of patients with TRD. This secondary analysis included patients aged 18–64 years who were treated/dosed according to USPI. The primary endpoint was remission, defined as Montgomery–Åsberg Depression Rating Scale (MADRS) total score ≤ 10. Treatment-emergent adverse events (TEAEs) leading to discontinuation were summarized descriptively.

**Results:**

Among 636 patients in this secondary analysis (ESK, n = 316; quetiapine XR, n = 320), significantly more ESK-treated patients achieved remission starting at week 8 (28.3% versus 18.6%; *P* = 0.005) through week 32 (55.7% versus 36.3%; *P* < 0.001), compared with quetiapine XR–treated patients. There were clinically and statistically significant improvements in MADRS scores with ESK versus quetiapine XR at each visit from day 8 onwards. Fewer patients discontinued treatment because of TEAEs with ESK (4.5%) versus quetiapine XR (10.1%).

**Conclusions:**

Consistent with the primary analysis, this secondary analysis demonstrated that ESK improves short- and long-term outcomes compared with quetiapine XR in patients with TRD treated according to USPI.

## Introduction

Major depressive disorder (MDD) is a highly prevalent, chronic condition associated with significant disease burden and economic costs.^1^ ^–^^4^ The optimal goal of treatment for patients with MDD is to achieve full remission, followed by maintenance treatment to avoid relapse.^5^ A substantial proportion of patients with MDD experience an inadequate response to multiple antidepressant interventions, resulting in a diagnosis of treatment-resistant depression (TRD).^6^ Although there is no consensus definition of TRD, a commonly used definition of TRD is an inadequate response to two or more oral antidepressants (OADs) despite adequate dose and adherence to treatment.^6^ ^,^^7^ TRD is estimated to affect 10% to 30% of patients with MDD.^1^ ^,^^3^ ^,^^6^ Based on international epidemiological estimates, more than 100 million people worldwide meet one or more definitions of TRD.^6^ ^,^^8^ TRD is associated with increased hospitalization, comorbidities, mortality, risk of suicide, and economic burden.^1^ ^–^^4^ ^,^^9^ ^,^^10^ The Sequenced Treatment Alternatives to Relieve Depression (STAR*D) trial demonstrated the importance of effective treatments in early lines of therapy to achieve remission, with the achievement of remission becoming less likely with every subsequent treatment step.^11^

Esketamine nasal spray (ESK) is a noncompetitive *N*-methyl-d-aspartate receptor antagonist approved by the US Food and Drug Administration for use in combination with an OAD for the treatment of adults with TRD.^12^ ESK has been shown to reduce depressive symptoms and risk of relapse versus placebo when given in combination with an OAD.^10^ ^,^^13^ ^,^^14^ In addition to clinical trial data, real-world studies have shown that ESK is well-tolerated and efficacious in improving depression symptoms.^15^ ^–^^17^Quetiapine extended-release (XR) is an atypical antipsychotic indicated for the treatment of MDD as an adjunct to OADs.^18^

Although the efficacy of ESK in TRD is well established, few studies have compared it with other treatments for TRD. Most analyses in previous studies have been retrospective, such as recent comparisons of ESK with repetitive transcranial magnetic stimulation and intravenous ketamine.^17^ ^,^^19^ Head-to-head studies are needed to compare the efficacy of ESK with that of other treatment options for TRD.

ESCAPE-TRD (NCT04338321) was a randomized, open-label, rater-blinded, long-term, phase 3b trial comparing ESK versus quetiapine XR in patients with TRD.^20^ Patients were randomly assigned to receive flexible doses of either ESK or quetiapine XR in combination with an ongoing OAD, according to the European Medicines Agency (EMA) summary of product characteristics.^21^ The primary endpoint was achieving remission, defined as a score of 10 or less on the Montgomery–Åsberg Depression Rating Scale (MADRS), at week 8. The key secondary endpoint was no relapse through week 32 after remission at week 8. The study’s primary endpoint and key secondary endpoints were met, demonstrating a benefit for ESK compared with quetiapine XR for the treatment of patients with TRD who had achieved remission at week 8. Safety data were in line with the established safety profiles of ESK and quetiapine XR, with no new safety signals identified.

This secondary analysis of the ESCAPE-TRD study evaluated the effects of ESK versus quetiapine XR in adult patients with TRD who received treatment according to US prescribing information. Specifically, patients who received ESK dosing of 28 mg were excluded from this analysis. To expand upon the data that have previously been published,^20^ several sensitivity analyses using multiple definitions of remission, relapse, time to remission, and response have been included to increase the robustness of the original analysis and aid in reassessing the primary and key secondary endpoints in this subpopulation. While the original study population received treatment according to EMA prescribing information, patients whose treatment followed the recommended US prescribing information were selected for this analysis, making this analysis of greater value for healthcare providers, patients, and decision-makers in the US and providing guidance to ensure the safe, effective, and appropriate administration of ESK.

## Methods

### Study design and patients

ESCAPE-TRD was a randomized, open-label, rater-blinded, long-term, phase 3b trial conducted across 171 sites in 24 countries.^20^ An open-label, pragmatic design was required owing to the differences in ESK and quetiapine XR administration. MADRS assessments were performed by an independent on-site rater blinded to the patient’s treatment and not involved in any other study assessments or treatment decisions.

Eligible patients had a history of ≥1 episode of MDD without evidence of response (<25% improvement) to ≥2 consecutive, adequately dosed treatments from ≥2 different antidepressant pharmacologic classes (including the ongoing treatment) during the current depressive episode. Randomization was performed with the use of a computer-generated schedule prepared before the trial in randomly permuted blocks and with stratification by age and number of previous treatment failures. The treatment period consisted of an 8-week acute phase followed by a 24-week maintenance phase (Figure S1 in the Supplementary Material). Patients included in this secondary analysis were aged 18–64 years and randomly assigned 1:1 to either ESK (56 or 84 mg) or quetiapine XR, both flexibly dosed (consistent with US prescribing information),^12^ ^,^^20^ in combination with an ongoing selective serotonin reuptake inhibitor or serotonin-norepinephrine reuptake inhibitor. ESK was dosed twice weekly (56 mg on day 1; may be increased to 84 mg from day 4) from week 1 to 4, weekly (56 or 84 mg) from week 5 to 8, and weekly or once every two weeks (56 or 84 mg) from week 9 to 32. Quetiapine XR was dosed daily, starting at 50 mg and titrated up to ≥150 mg/day by the end of week 2, and was then flexibly dosed (150–300 mg/day) from week 3 to 32. The trial ended per protocol after all participants completed the final follow-up visit.

This study was approved by country-specific ethics review boards and conducted in accordance with the ethical principles of the Declaration of Helsinki. All patients provided written informed consent.

### Endpoints and statistical analysis

Demographics and efficacy endpoint analyses included all randomly assigned patients. The primary endpoint was the proportion of patients achieving remission at week 8, defined as MADRS total score ≤ 10. The key secondary endpoint was the proportion of patients who were relapse-free through week 32 (without treatment discontinuation) after achieving remission at week 8. A relapse was defined as any of the following: worsening of depressive symptoms as indicated by MADRS total score ≥ 22, confirmed by one additional assessment of MADRS within the next 5–15 days; any psychiatric hospitalization for worsening of depression or suicide prevention or due to a suicide attempt; or suicide attempt, completed suicide, or any other clinically relevant event determined per the investigator’s clinical judgment to be indicative of a relapse of depressive illness but for which the patient was not hospitalized. The primary and key secondary endpoints were compared between groups using a Cochran–Mantel–Haenszel test adjusting for a total number of treatment failures (2; ≥3). Patients who discontinued treatment were considered to have a negative outcome for the primary and key secondary endpoints. All endpoints were tested at a two-sided 0.05 significance level without adjustment for multiple testing.

The proportion of patients with remission (MADRS total score ≤ 10) or response (≥50% reduction in MADRS total score or MADRS total score ≤ 10) at each on-treatment visit were compared between the treatment groups using a last observation carried forward (LOCF) approach with a Cochran–Mantel–Haenszel test. Odds ratios (ORs) were calculated to compare the effect of ESK + OAD and quetiapine XR + OAD on remission and response outcomes as measured by the MADRS scale. MADRS change from baseline (CFB) between study arms was analyzed using mixed models for repeated measures (with an unstructured covariance matrix). The model included the baseline MADRS score as a covariate and treatment, stratification factors, visit, and visit-by-treatment interaction terms as fixed effects.

Sensitivity analyses were prespecified on the primary and secondary endpoints by varying parameters in their definitions, namely the time points, thresholds, and scales used. For sensitivity analyses based on alternative thresholds, the threshold for remission was adjusted to MADRS total score ≤ 12 (to provide comparisons with previous studies^13^ ^,^^14^) and MADRS total score ≤ 8, and the threshold for relapse was adjusted to MADRS total score ≥ 18. An alternative analysis considered a Clinical Global Impression-Severity scale (CGI-S) total score of ≥5 as the definition of relapse. For sensitivity analyses based on time points, the timepoint for achieving remission was adjusted to week 6, week 10, and any point within 8 weeks, with the definition of remission unchanged. Rates of remission over time-based on alternative thresholds for remission were also assessed between the treatment groups.

Time to first remission was defined as the duration of time elapsed from baseline to the visit at which the patient achieved a MADRS total score ≤ 10. Time to first response was defined as the duration of time until a ≥ 50% improvement from baseline in MADRS total score, or MADRS total score ≤ 10 was reported. Time to confirmed remission and response was defined as the time to the first occurrence of achieving remission or response at two consecutive visits. Time to MADRS remission and response were analyzed using the Kaplan–Meier method. Observed data from patients were included in the analyses for as long as patients remained on study treatment. Patients who dropped out or discontinued study intervention were censored at the time of discontinuation and assumed to have never achieved the event.

The safety analysis included all patients who received at least one dose of any study intervention. Safety evaluations were performed throughout the trial. An adverse event was counted as treatment-emergent if it started after taking the first dose and on or before 14 days after the last dose of study medication. Serious adverse events were considered treatment-emergent if they started within 30 days of the last dose.^20^

## Results

### Patient characteristics

A total of 676 patients were included in the overall study between August 26, 2020, and November 5, 2021. Of these, 636 were included in this secondary analysis, with 316 patients randomly assigned to the ESK arm and 320 patients to the quetiapine XR arm (Figure S2 in the Supplementary Material). The safety analysis set included 314 patients in the ESK arm and 316 patients in the quetiapine XR arm. Baseline characteristics were comparable between study arms (Table 1). The mean age was 43.7 years, and 65.9% of patients were female. Mean disease severity scores at baseline were similar between groups and consistent with moderate-to-severe depressive symptoms.

**Table 1.** Demographic and Clinical Characteristics at Baseline

	**Quetiapine XR + OAD** ***n* = 320**	**ESK + OAD** ***n* = 316**	**Total** ***N* = 636**
Age, years			
Mean (SD)	44.5 (12.40)	42.8 (12.56)	43.7 (12.50)
Median (range)	46.0 (18 to 64)	44.0 (18 to 64)	45.0 (18 to 64)
Sex, *n* (%)			
Male	113 (35.3)	104 (32.9)	217 (34.1)
Female	207 (64.7)	212 (67.1)	419 (65.9)
Body mass index, kg/m^2^, *n* (%)^a^			
<18.5 (underweight)	5 (1.9)	6 (2.3)	11 (2.1)
18.5 to <25 (normal)	84 (31.1)	105 (39.9)	189 (35.5)
25 to <30 (overweight)	93 (34.4)	91 (34.6)	184 (34.5)
≥30 (obese)	88 (32.6)	61 (23.2)	149 (28.0)
Employment status, *n* (%)			
Employed	175 (54.7)	177 (56.0)	352 (55.3)
Unemployed	145 (45.3)	138 (43.7)	283 (44.5)
Other	0	1 (0.3)	1 (0.2)
Treatment failures, *n* (%)			
2	192 (60.0)	187 (59.2)	379 (59.6)
≥3	128 (40.0)	129 (40.8)	257 (40.4)
Age when diagnosed with MDD, years			
Mean (SD)	34.4 (11.67)	33.0 (11.58)	33.7 (11.64)
Median (range)	34.5 (10 to 55)	32.0 (10 to 54)	33.0 (10 to 55)
Baseline MADRS total score			
Mean (SD)	31.1 (5.94)	31.6 (6.08)	31.3 (6.01)
Median	31.0 (12 to 51)	32.0 (6 to 52)	31.0 (6 to 52)
Baseline IDS-C30 total score			
Mean (SD)	45.2 (6.90)	44.8 (6.65)	45.0 (6.77)
Median (range)	45.0 (28 to 71)	44.0 (17 to 66)	45.0 (17 to 71)
Baseline CGI-S score			
Mean (SD)	4.9 (0.71)	4.9 (0.62)	4.9 (0.67)
Median (range)	5.0 (3 to 6)	5.0 (3 to 7)	5.0 (3 to 7)

Abbreviations: CGI-S, Clinical Global Impression-Severity scale; ESK, esketamine nasal spray; IDS-C30, Inventory of Depressive Symptomatology – Clinician-rated, 30-item scale; MADRS, Montgomery–Åsberg Depression Rating Scale; MDD, major depressive disorder; OAD, oral antidepressant; XR, extended-release.

a Denominators for quetiapine XR: *n* = 270; ESK, *n* = 263; total, *N* = 533.

### Primary and secondary endpoints

**Figure 1.**
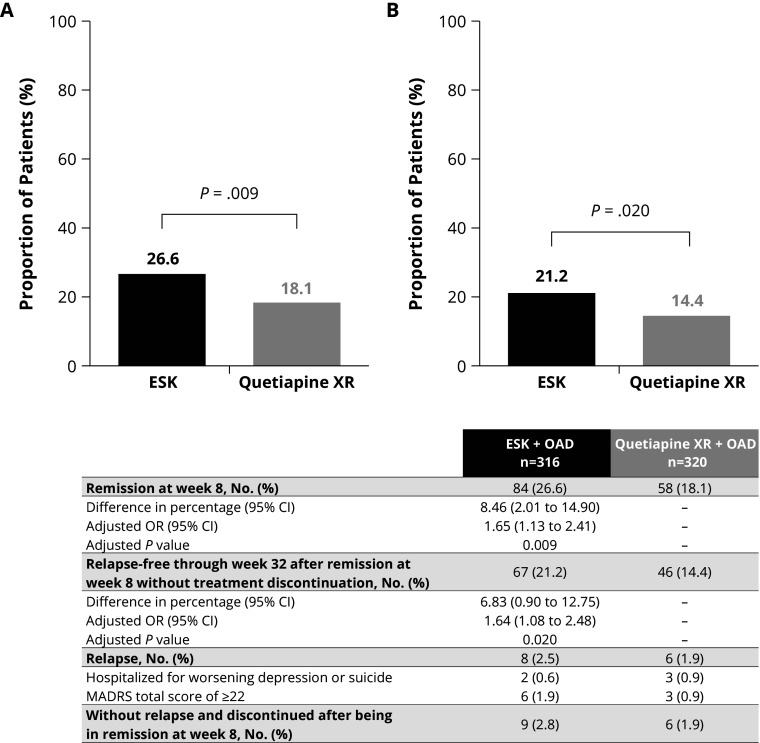
Proportion of patients (A) who achieved remission at week 8 and (B) remained relapse-free through week 32 after remission at week 8 without treatment discontinuation. Abbreviations: CI, confidence interval; ESK, esketamine nasal spray; MADRS, Montgomery–Åsberg Depression Rating Scale; OAD, an oral antidepressant; OR, odds ratio; XR, extended-release.

Remission rates without treatment discontinuation were significantly higher at week 8 with ESK versus quetiapine XR (26.6% versus 18.1%; *P* = 0.009) (Figure 1A). The odds of achieving remission at week 8 with ESK + OAD were 1.65 times higher (OR: 1.65; 95% CI, 1.13–2.41) than with quetiapine XR + OAD. Furthermore, a significantly greater proportion of patients were relapse-free through week 32 after remission at week 8 without treatment discontinuation with ESK versus quetiapine XR (21.2% versus 14.4%, respectively; *P* = 0.020) (Figure 1B). Approximately 80% of patients in both groups who achieved remission at week 8 remained relapse-free through week 32 (ESK, 79.8%; quetiapine XR, 79.3%).

### Remission and response rates over time

**Figure 2.**
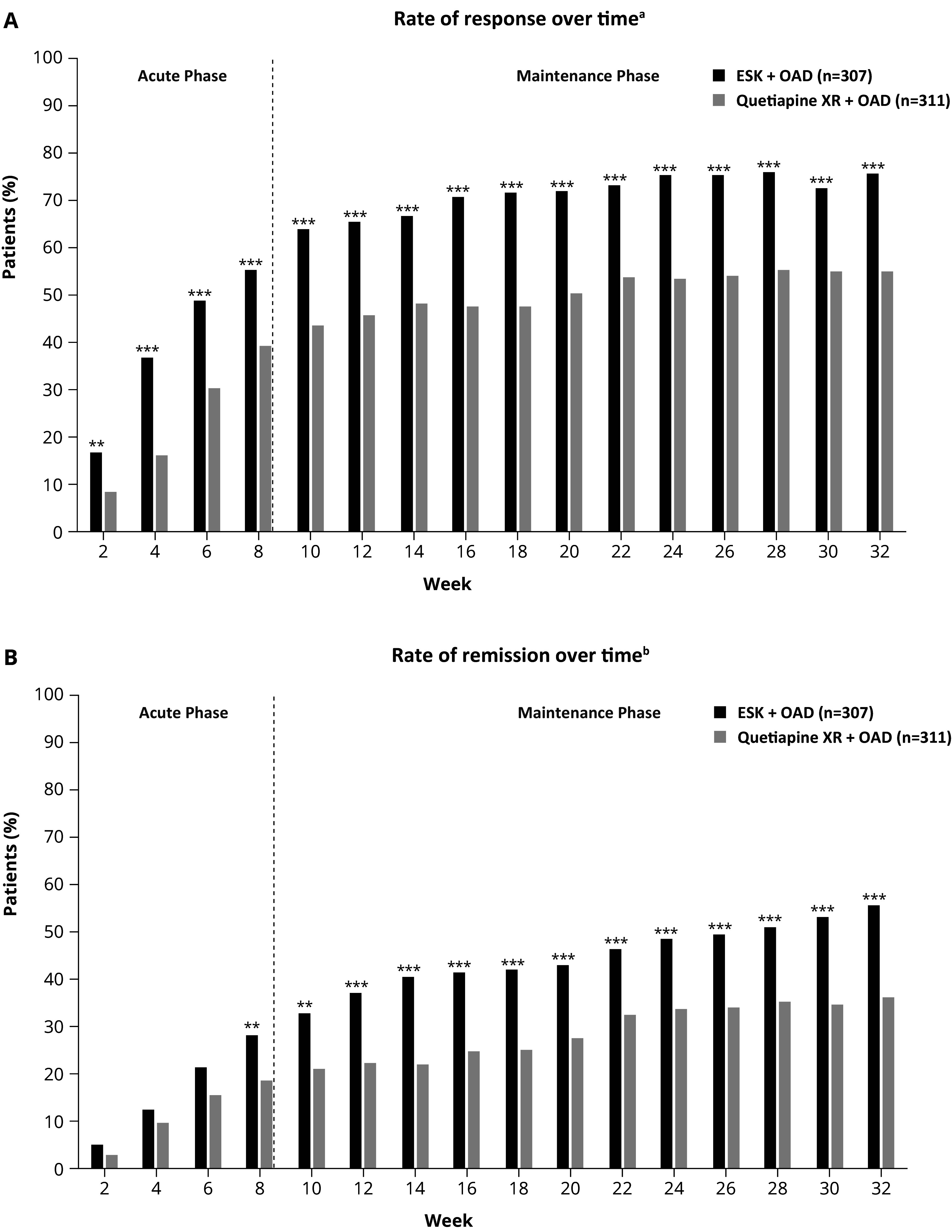
(A) Response and (B) remission rates over time (LOCF). The full analysis set includes all randomly assigned patients. Percentages are based on the number of patients at each timepoint, using LOCF for missing data. Data for weeks 2 and 4 correspond to days 15 and 29, respectively. Abbreviations: ESK, esketamine nasal spray; LOCF, last observation carried forward; MADRS, Montgomery–Åsberg Depression Rating Scale; OAD, oral antidepressant; XR, extended-release. ^a^Response was defined as ≥50% improvement in MADRS total score or MADRS total score ≤ 10. Testing was done with a 2-sided 0.05 significance level without adjustment for multiple testing. ^b^Remission was defined as a MADRS total score ≤ 10. **P < 0.01; ***P < 0.001.

A significantly higher percentage of patients from the ESK arm experienced response starting at week 2 (16.6% versus 8.4%, *P* = 0.002) and at every subsequent time point through week 32 (75.9% versus 55.0%, *P* < .001) (Figure 2A) compared with the quetiapine XR arm. The odds of achieving response at week 32 for the ESK arm were 2.6 times higher than for the quetiapine XR arm (OR: 2.58; 95% CI, 1.83–3.64). A higher percentage of patients in the ESK arm achieved remission starting at week 8 and at every subsequent time point through week 32 compared with those in the quetiapine XR arm (Figure 2B). The absolute rates of remission at week 8 with ESK and quetiapine XR were 28.3% and 18.6%, respectively (*P* = 0.005); at week 32, remission rates were 55.7% and 36.3%, respectively (*P* < 0.001). The odds of achieving remission at week 32 for the ESK arm were 2.2 times higher than for the quetiapine XR arm (OR: 2.20; 95% CI, 1.60–3.04). There were clinically and statistically significant improvements in MADRS scores with ESK compared with quetiapine XR at each visit from day 8 onward, with an average difference over time in the least squares means total MADRS score CFB of −2.5 (95% CI, −3.5 to −1.4) (Figure S3 and Table S1 in the Supplementary Material).

### Sensitivity analyses of the primary and secondary endpoints

For all sensitivity analyses using alternative definitions of remission, the proportion of patients achieving the primary endpoint was higher for patients receiving ESK than for those receiving quetiapine XR (Figure S4 in the Supplementary Material). When using a remission cut-off of MADRS total score ≤ 12 (as was used in prior ESK studies^13^), the remission rates at week 8 with ESK and quetiapine XR were 38.3% and 23.4%, respectively (*P* < 0.001). For a remission cut-off of MADRS total score ≤ 8, the remission rates at week 8 with ESK and quetiapine XR were 17.4% and 13.1%, respectively (*P* = 0.131). With a remission cut-off of MADRS total score ≤ 10, the remission rates with ESK and quetiapine XR at week 6 were 20.3% and 15.0%, respectively. At week 10, the remission rates with ESK and quetiapine XR were 31.0% and 20.3%, respectively. A total of 34.5% of patients receiving ESK versus 20.9% receiving quetiapine XR achieved remission at any point within the first 8 weeks.

Similarly, for all sensitivity analyses using alternative definitions of remission and relapse, the proportion of patients achieving the key secondary endpoint was higher for patients receiving ESK than for those receiving quetiapine (Figure S5 in the Supplementary Material). When adjusting the cutoff to a MADRS total score ≤ 12, 32.0% of patients receiving ESK achieved remission at week 8 and remained relapse-free through week 32, compared with 17.8% of patients receiving quetiapine XR (*P* < 0.001). For a remission cut-off of MADRS total score ≤ 8, the percentage of patients who remained relapse-free through week 32 after achieving remission at week 8 with ESK and quetiapine XR were 14.2% and 10.0%, respectively (*P* = 0.093). When considering a CGI-S total score of ≥5 as the definition of relapse, 22.8% of patients receiving ESK achieved remission at week 8 and remained relapse-free through week 32 versus 14.7% of patients receiving quetiapine XR (*P* = 0.008).

### Remission over time using cut-offs of MADRS ≤ 12 and ≤ 8

Rates of remission over time based on alternative thresholds for remission were analyzed for all randomly assigned patients using an LOCF approach. A greater proportion of patients treated with ESK achieved remission with a cut-off of MADRS total score ≤ 12 at each timepoint, from week 2 through to week 32, compared with patients treated with quetiapine XR (Figure S6 in the Supplementary Material). At week 8, 40.4% (*n* = 124) of patients in the ESK arm achieved remission, compared with 24.4% (*n* = 76) of patients in the quetiapine XR arm (risk ratio (RR): 1.66; 95% CI, 1.31–2.10; *P* < 0.001). At week 32, 65.1% (*n* = 200) of patients receiving ESK achieved remission, compared with 46.3% (*n* = 144) of patients receiving quetiapine XR (RR: 1.42; 95% CI, 1.23–1.64; *P* < 0.001). Similarly, for a remission cut-off of MADRS total score ≤ 8, a greater proportion of patients treated with ESK achieved remission at most timepoints from week 2 through to week 32, compared with patients treated with quetiapine XR. At week 8, 18.6% (*n* = 57) of patients in the ESK arm achieved remission, compared with 13.5% (*n* = 42) of patients in the quetiapine XR arm (RR: 1.37; 95% CI, 0.95–1.98; *P* = 0.090). At week 32, 42.7% (*n* = 131) of patients receiving ESK achieved remission, compared with 26.7% (*n* = 83) of patients receiving quetiapine XR (RR: 1.62; 95% CI, 1.29–2.03; *P* < 0.001).

### Time to first and confirmed remission and response

**Figure 3.**
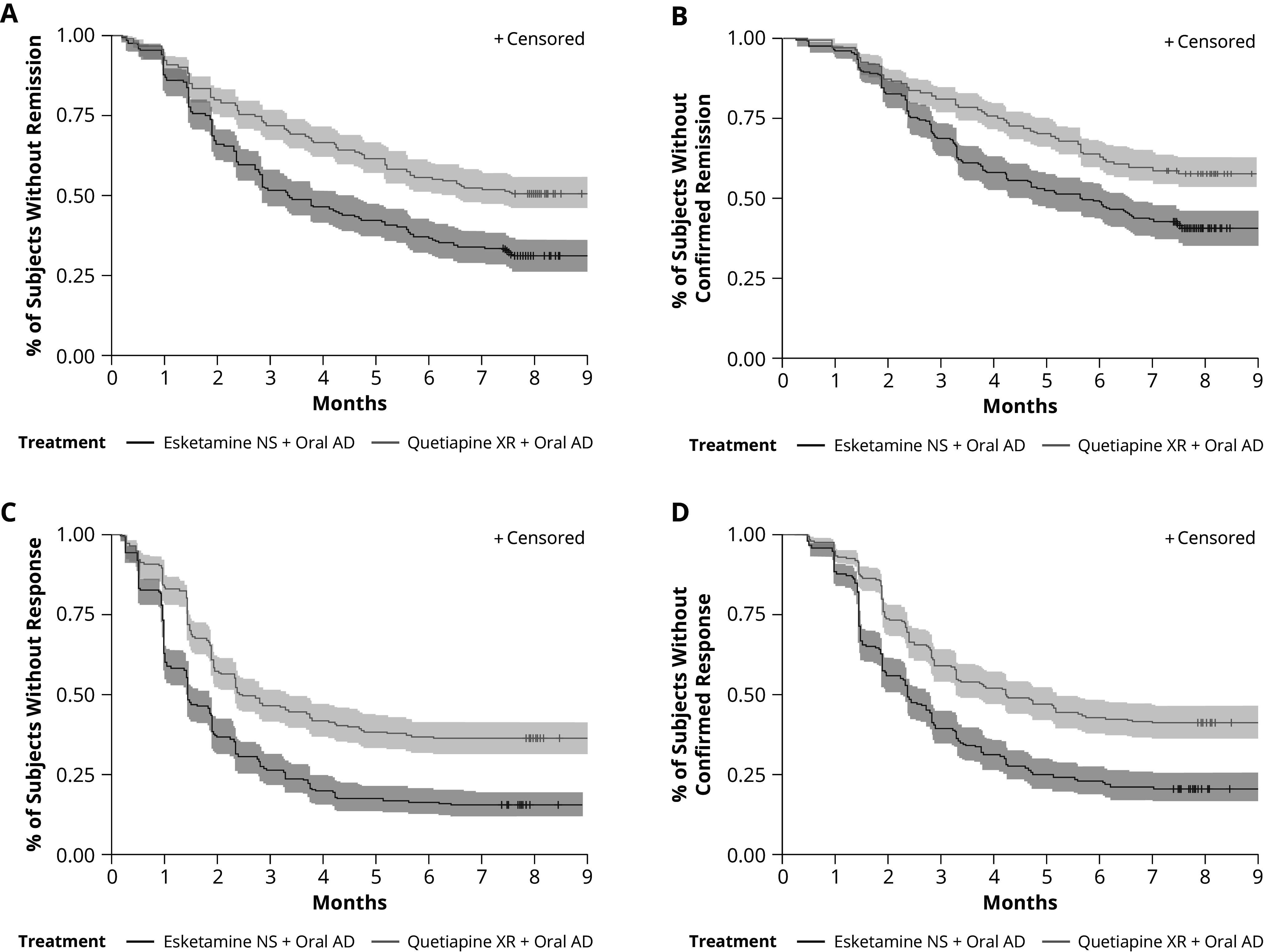
Kaplan Meier plot of time to MADRS (A) first and (B) confirmed remission and (C) first and (D) confirmed response. Abbreviations: AD, antidepressant; MADRS, Montgomery–Åsberg Depression Rating Scale; NS, nasal spray; XR, extended-release. Remission was defined as a MADRS total score ≤ 10. The response was defined as ≥50% improvement in MADRS total score or MADRS total score ≤ 10. Confirmed remission and response were defined as the time to the first occurrence of achieving remission or response at two consecutive visits.

Treatment with ESK shortened the time to first remission versus quetiapine XR (Figure 3A); time to confirmed remission, defined as achieving remission at two consecutive visits, was also shortened with ESK treatment (Figure 3B). Similarly, ESK treatment shortened both the time to first response and time to confirmed response (achieving response at two consecutive visits) compared with quetiapine XR (Figure 3C, D).

### Safety

Of the 630 patients included in the safety analysis set, 289 of 314 patients (92.0%) in the ESK arm and 248 of 316 (78.5%) in the quetiapine XR arm experienced at least one treatment-emergent adverse event (TEAE) (Table 2). Across both treatment groups, 5.4% of patients (*n* = 34) experienced at least one serious TEAE. The most common TEAEs (occurring in ≥10% of patients) were dizziness, headache, somnolence, nausea, dissociation, and vertigo.

**Table 2.** Summary of TEAEs

*n* (%)	**Quetiapine XR + OAD** ***n* = 316**	**ESK + OAD** ***n* = 314**	**Total** ***N* = 630**
≥1 TEAE	248 (78.5)	289 (92.0)	537 (85.2)
TEAE possibly related to treatment	197 (62.3)	266 (84.7)	463 (73.5)
TEAE leading to death	0	1 (0.3)	1 (0.2)
≥1 serious TEAE	15 (4.7)	19 (6.1)	34 (5.4)
TEAE leading to study drug withdrawn	32 (10.1)	14 (4.5)	46 (7.3)
TEAE leading to dose interruption/reduction	39 (12.3)	32 (10.2)	71 (11.3)
**TEAEs in at least 5% of subjects in either treatment group**			
Nervous system disorders	149 (47.2)	218 (69.4)	367 (58.3)
Dizziness	25 (7.9)	148 (47.1)	173 (27.5)
Headache	41 (13.0)	80 (25.5)	121 (19.2)
Somnolence	74 (23.4)	47 (15.0)	121 (19.2)
Sedation	27 (8.5)	20 (6.4)	47 (7.5)
Paraesthesia	2 (0.6)	37 (11.8)	39 (6.2)
Dysgeusia	1 (0.3)	36 (11.5)	37 (5.9)
Hypoesthesia	1 (0.3)	17 (5.4)	18 (2.9)
Gastrointestinal disorders	62 (19.6)	134 (42.7)	196 (31.1)
Nausea	10 (3.2)	94 (29.9)	104 (16.5)
Vomiting	4 (1.3)	35 (11.1)	39 (6.2)
Dry mouth	21 (6.6)	3 (1.0)	24 (3.8)
Psychiatric disorders	44 (13.9)	149 (47.5)	193 (30.6)
Dissociation	2 (0.6)	89 (28.3)	91 (14.4)
Confusional state	1 (0.3)	19 (6.1)	20 (3.2)
Infections and infestations	65 (20.6)	68 (21.7)	133 (21.1)
COVID–19	27 (8.5)	24 (7.6)	51 (8.1)
Nasopharyngitis	10 (3.2)	20 (6.4)	30 (4.8)
General disorders and administration site conditions	53 (16.8)	63 (20.1)	116 (18.4)
Fatigue	34 (10.8)	18 (5.7)	52 (8.3)
Investigations	51 (16.1)	47 (15.0)	98 (15.6)
Weight increased	39 (12.3)	9 (2.9)	48 (7.6)
Blood pressure increased	4 (1.3)	24 (7.6)	28 (4.4)
Ear and labyrinth disorders	5 (1.6)	65 (20.7)	70 (11.1)
Vertigo	3 (0.9)	61 (19.4)	64 (10.2)
Musculoskeletal and connective tissue disorders	24 (7.6)	39 (12.4)	63 (10.0)
Back pain	8 (2.5)	16 (5.1)	24 (3.8)
Respiratory, thoracic, and mediastinal disorders^a^	9 (2.8)	51 (16.2)	60 (9.5)
Eye disorders	5 (1.6)	29 (9.2)	34 (5.4)
Vision blurred	3 (0.9)	19 (6.1)	22 (3.5)
Metabolism and nutrition disorders	20 (6.3)	9 (2.9)	29 (4.6)
Skin and subcutaneous tissue disorders	8 (2.5)	18 (5.7)	26 (4.1)

Abbreviations: AE, adverse event; COVID-19, coronavirus disease 2019; ESK, esketamine nasal spray; OAD, oral antidepressant; TEAE, treatment-emergent adverse event; XR, extended-release.

An AE was considered a TEAE if it started between the first dose and the safety follow-up visit (14 days after last dose of study treatment) or ≤ 30 days after last dose (for serious AEs).

a There were no reported cases of respiratory depression or oxygen saturation decreased.

Treatment discontinuation occurred in 22.2% of patients in the ESK arm and 40.0% in the quetiapine XR arm. Fewer patients treated with ESK (4.5%) discontinued treatment due to TEAEs compared with those treated with quetiapine XR (10.1%). TEAEs most commonly leading to treatment discontinuation were sedation (quetiapine XR, *n* = 6), weight increase (quetiapine XR, *n* = 5), dizziness (ESK, *n* = 2; quetiapine XR, *n* = 4), and fatigue (quetiapine XR, *n* = 4).

## Discussion

This secondary analysis of the ESCAPE-TRD study directly compared the efficacy of ESK with quetiapine XR in adult patients with TRD receiving an ongoing OAD who received treatment in accordance with US prescribing information. Our findings were consistent with the results of the total study population.^20^ Because the population included in this analysis represents practice according to the US prescribing information (the original study population received treatment according to EMA prescribing information), these results further support the benefits of ESK compared with quetiapine XR and provide valuable guidance to clinicians, patients, and decision-makers in the US to ensure the safe, effective, and appropriate administration of ESK.

Remission rates without treatment discontinuation were significantly higher at week 8 with ESK versus quetiapine XR (26.6% versus 18.1%; *P* = 0.009). Furthermore, a significantly greater proportion of patients in the ESK arm were relapse-free through week 32 after remission at week 8, without treatment discontinuation versus quetiapine XR (21.2% versus 14.4%, respectively; *P* = 0.020). ESK, in combination with an OAD, was associated with an increase in the proportion of patients achieving response and remission over time compared with quetiapine XR in combination with an OAD. Change from baseline over time in MADRS total score was significantly greater in the ESK arm than in the quetiapine XR arm.

The results presented herein are in accordance with real-world evidence from the ICEBERG study.^22^ ICEBERG was an adjusted indirect treatment comparison estimating the long-term benefit of ESK when compared with routine real-world treatment (RWT) of TRD in general psychiatry. ICEBERG supported that, over 6 months, ESK demonstrated statistically significant benefit over RWT for patients with TRD in achieving both response and remission.

While remission and prevention of relapse are the main therapeutic goals for treating depression, there is a lack of consensus regarding how remission and relapse are defined. For all sensitivity analyses, however, patients treated with ESK were more likely to achieve both the primary and key secondary endpoints than patients treated with quetiapine XR. Remission rates continued to increase from week 8 to week 32 in both treatment arms, with a greater proportion of patients in the ESK arm achieving remission regardless of the remission definition.

Safety data from this secondary analysis were consistent with the overall study population and the known safety profiles of each treatment, with no new safety signals identified. TEAEs were reported at a higher incidence with ESK than with quetiapine XR; however, the rates of treatment discontinuation due to TEAEs were generally lower with ESK + OAD than with quetiapine XR + OAD. The majority of ESK TEAEs, including dizziness, nausea, dissociation, and vertigo, were transient and resolved on the same day as dosing, usually while patients were still under clinical supervision.^23^ In contrast, the most common TEAEs experienced with quetiapine XR treatment, including fatigue and weight increase, tended to be chronic in nature and were therefore possibly more likely to contribute to treatment discontinuation.^23^ Of note, weight increase, a common occurrence with psychiatric medications,^24^ was more common in the quetiapine XR arm than in the ESK arm (12.3% versus 2.9%, respectively).

The results of this study must be interpreted within its limitations. Firstly, differences in treatment adherence and routes of administration could potentially introduce bias in the results. Because the routes of administration were different, an open-label design was selected to eliminate the need for a placebo and minimize patient burden. The open-label design better reflected real-world practice because it permitted treatment administration according to product labels. An additional potential confounder was that the two groups had different frequencies and durations of study visits. Because ESK must be administered under the supervision of a healthcare professional, patients in the ESK arm had twice-weekly visits for the first 4 weeks of the study, in line with real-world practice. During the same period, patients in the quetiapine XR arm had once-weekly visits, which is more frequent than typical clinical practice. It should also be noted that engagement with healthcare professionals was higher for both arms than is typical for treatment with OADs only.

In conclusion, consistent with the primary analysis, results from this secondary analysis demonstrated that ESK improves short- and long-term outcomes compared with quetiapine XR in patients with TRD treated according to US prescribing information.

## Supporting information

McIntyre et al. supplementary material McIntyre et al. supplementary material

## Supplementary material

The supplementary material for this article can be found at http://doi.org/10.1017/S1092852924002451.

## Access to data

All authors had full access to all the data in the study and take responsibility for the integrity of the data and the accuracy of the data analysis.

## Data sharing statement

The data sharing policy of Janssen Pharmaceutical Companies of Johnson & Johnson is available at https://www.janssen.com/clinical-trials/transparency. As noted on this site, requests for access to the study data can be submitted through the Yale Open Data Access (YODA) Project site at http://yoda.yale.edu.
