# Supplementary material for: Esketamine nasal spray versus quetiapine XR in adults with treatment-resistant depression: a secondary analysis of the ESCAPE-TRD randomized clinical trial
Source: CNS Spectr. 2025 Jan 17;30(1):e26. doi: 10.1017/S1092852924002451 (PMC13064706; doi:10.1017/S1092852924002451)
Supplement: McIntyre et al. supplementary material [file S1092852924002451sup001.zip › 150205801_ESCAPE TRD CFL Graphical Abstract_Final.pdf]

Esketamine Nasal Spray Versus Quetiapine XR in Adults With Treatment-Resistant Depression: A Secondary Analysis of the ESCAPE-TRD Randomized Clinical Trial

What is treatment-resistant depression?

Approximately 10%-30% of patients with major depressive disorder experience an inadequate response to multiple antidepressants, leading to a diagnosis of treatment-resistant depression (TRD)

TRD is associated with increased hospitalization, comorbidities, mortality, risk of suicide, and economic burden

Esketamine nasal spray (ESK) is approved by the United States Food and Drug Administration for use in combination with an oral antidepressant for the treatment of adults with TRD

What did this study look at?

ESCAPE-TRD was a randomized, open-label, rater-blinded, phase 3b trial investigating ESK versus quetiapine extended-release (XR) for acute and maintenance treatment of patients with TRD. Rates of remission and response and improvements in depressive symptoms over time were compared between adult patients with TRD who received ESK and those who received quetiapine XR, both in accordance with US prescribing information

What did this study find?

636 patients were included in this secondary analysis

ESK 316

Quetiapine XR 320

Mean age of 43.7 years

65.9% female

Montgomery–Åsberg Depression Rating Scale (MADRS) scores significantly improved with ESK versus quetiapine XR at each visit from day 8 onwards

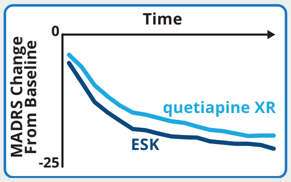

- Significantly more patients achieved response (≥50% improvement in MADRS score or MADRS score ≤10) with **ESK** versus **quetiapine XR**

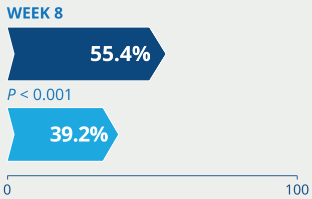

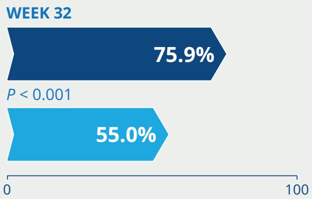

- Significantly more patients achieved remission (MADRS score ≤10) with **ESK** versus **quetiapine XR**

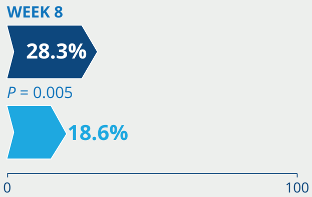

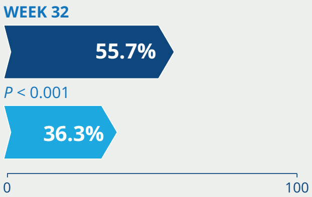

- Fewer patients treated with ESK discontinued treatment because of treatment-emergent adverse events

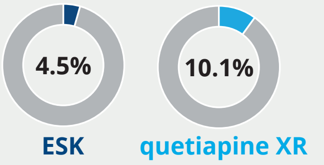

What do these findings mean?

ESK improves short- and long-term outcomes in patients with TRD treated according to US prescribing information compared to those treated with quetiapine XR
