# Supplementary material for: Esketamine nasal spray versus quetiapine XR in adults with treatment-resistant depression: a secondary analysis of the ESCAPE-TRD randomized clinical trial
Source: CNS Spectr. 2025 Jan 17;30(1):e26. doi: 10.1017/S1092852924002451 (PMC13064706; doi:10.1017/S1092852924002451)
Supplement: McIntyre et al. supplementary material [file S1092852924002451sup001.zip › 150205803_ESCAPE-TRD CFL Plain Language Summary_Final.docx]

**Manuscript title**: Esketamine nasal spray versus quetiapine XR in adults with treatment-resistant depression: a secondary analysis of the ESCAPE-TRD randomized clinical trial

**Plain Language Summary**:

Approximately 10%-30% of patients with major depressive disorder (MDD) are diagnosed with treatment-resistant depression (TRD), defined as an inadequate response to treatment with 2 or more types of oral antidepressants (OADs). Esketamine nasal spray (ESK), in combination with an OAD, is approved in the United States for the treatment of adults with TRD. ESK is also approved for the treatment of depressive symptoms in adults with MDD who have acute suicidal thoughts or behavior. The goal of MDD treatment is to achieve remission in symptoms without relapse. However, there is no consensus on how remission and relapse are defined.

The ESCAPE-TRD clinical trial (NCT04338321) compared ESK versus quetiapine extended-release (XR), an atypical antipsychotic used to treat MDD, in patients with TRD. The trial was conducted in 24 countries. This secondary analysis measured outcomes in patients treated according to US prescribing information (USPI), using a range of definitions for both remission and relapse.

A total of 636 patients were included in the analysis. Of these, 316 received ESK and 320 received quetiapine XR. Significantly more patients treated with ESK achieved remission compared with those treated with quetiapine XR regardless of how remission was defined.

When response was defined as a ≥50% reduction in Montgomery-Åsberg Depression Rating Scale [MADRS] total score or MADRS ≤10, a significantly higher proportion of patients treated with ESK achieved response starting at week 2 (16.6% vs. 8.4%, *P* = 0.002) through week 32 (75.9% vs. 55.0%, *P* < 0.001) of treatment versus patients treated with quetiapine XR. When remission was defined as a MADRS total score of ≤10, 28.3% of patients treated with ESK achieved remission at week 8 of treatment versus 18.6% of patients treated with quetiapine XR. At week 32, 55.7% of patients treated with ESK achieved remission versus 36.3% of patients treated with quetiapine XR. Furthermore, fewer patients treated with ESK versus quetiapine XR (4.5% vs 10.1%) discontinued treatment because of side effects that developed during treatment.

Overall, the results of this analysis of patients treated according to USPI are consistent with those of the total study population and demonstrate that ESK improves short- and long-term outcomes compared to quetiapine XR. Because the population included in this analysis represents real-world clinical practice, these results further support the benefits of ESK compared to quetiapine XR and provide valuable guidance to clinicians, patients, and decision makers for potential treatment goals.
