# Supplementary material for: Esketamine nasal spray versus quetiapine XR in adults with treatment-resistant depression: a secondary analysis of the ESCAPE-TRD randomized clinical trial
Source: CNS Spectr. 2025 Jan 17;30(1):e26. doi: 10.1017/S1092852924002451 (PMC13064706; doi:10.1017/S1092852924002451)
Supplement: McIntyre et al. supplementary material [file S1092852924002451sup001.zip › 150206062_ESCAPE-TRD CFL Supplementary Material_Final.docx]

Supplementary Material

Table S1. Estimated Difference in MADRS Change From Baseline Between ESK and Quetiapine XR at Each Visit

Figure S1. Study Design

Figure S2. CONSORT Diagram

Figure S3. LS Mean Change in MADRS Total Score Over Time

Figure S4. Proportion of Patients Achieving the Primary Endpoint per Sensitivity Analysis

Figure S5. Proportion of Patients Achieving the Key Secondary Endpoint per Sensitivity Analysis

Figure S6. Patients in Remission Based on MADRS Total Scores ≤8, ≤10, and ≤12 Over Time (LOCF)

Table S1. Estimated Difference in MADRS Change From Baseline Between ESK and Quetiapine XR at Each Visit

|  | **ESK – Quetiapine XR**  **MADRS CFB (95% CI)**  ***P* value** |
| --- | --- |
| Day 8 | −1.5 (−2.4 to −0.7)  < .001 |
| Day 15 | −2.9 (−3.9 to −1.8)  < .001 |
| Day 29 | −3.2 (−4.4 to −2.0)  < .001 |
| Week 6 | −3 (−4.3 to −1.7)  < .001 |
| Week 8 | −2.8 (−4.2 to −1.4)  < .001 |
| Week 10 | −3 (−4.3 to −1.7)  < .001 |
| Week 12 | −2.9 (−4.2 to −1.5)  < .001 |
| Week 14 | −2.9 (−4.3 to −1.6)  < .001 |
| Week 16 | −2.9 (−4.3 to −1.6)  < .001 |
| Week 18 | −2.6 (−4.1 to −1.2)  < .001 |
| Week 20 | −2 (−3.5 to −0.6)  .006 |
| Week 22 | −2.1 (−3.5 to −0.7)  .003 |
| Week 24 | −2 (−3.5 to −0.6)  .006 |
| Week 26 | −2 (−3.4 to −0.6)  .006 |
| Week 28 | −1.5 (−3.0 to −0.1)  .036 |
| Week 30 | −1.8 (−3.3 to −0.3)  .018 |
| Week 32 | −2.4 (−3.8 to −1.0)  < .001 |
| Overall | −2.5 (−3.5 to −1.4)  < .001 |

Abbreviations: ANCOVA, analysis of covariance; CFB, change from baseline; CI, confidence interval; ESK, esketamine nasal spray; MADRS, Montgomery‑Åsberg Depression Rating Scale; XR, extended-release.
Least-squares mean, standard error, 95% CI, and 2-sided *P* value are based on a repeated-measures mixed-effects ANCOVA model with treatment, number of treatment failure, time, time by treatment, and baseline value as covariates. They are modeled with an unstructured covariance structure.

Figure S1. Study Design

**
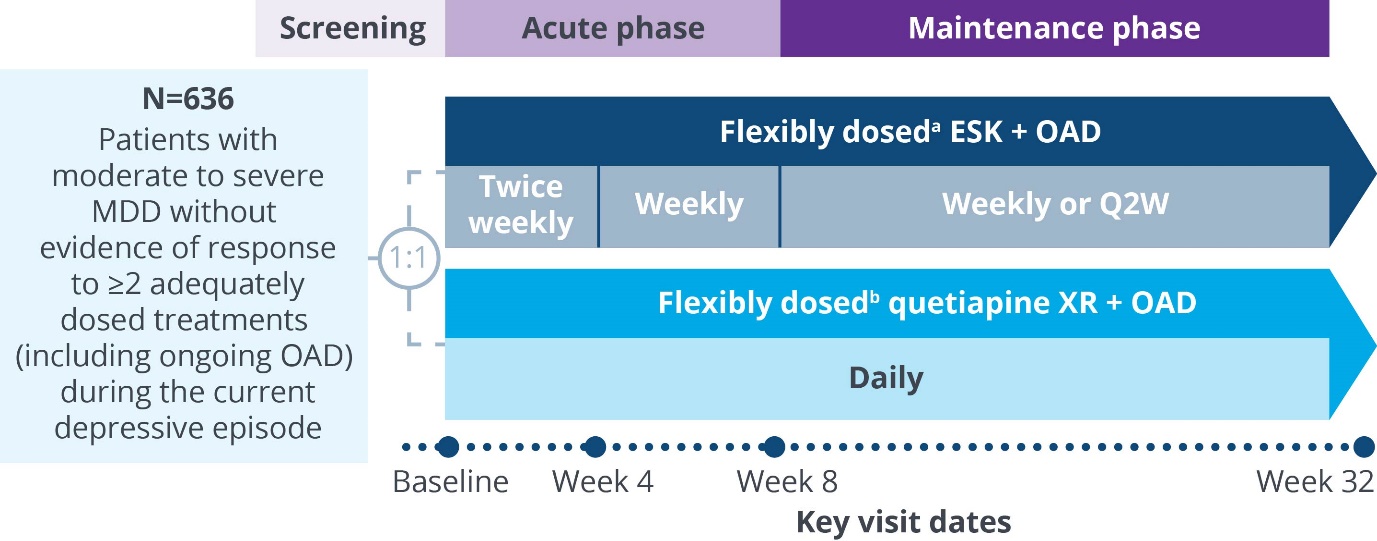
**

Abbreviations: ESK, esketamine nasal spray; MDD, major depressive disorder; OAD, oral antidepressant; Q2W, every 2 weeks; SNRI, serotonin norepinephrine reuptake inhibitors; SSRI, selective serotonin reuptake inhibitors; XR, extended-release.

OADs were SSRIs or SNRIs.

^a^ESK was dosed twice weekly (56 mg on day 1 and may be increased to 84 mg from day 4) through weeks 1 to 4, weekly (56 or 84 mg) through weeks 5 to 8, and weekly or Q2W (56 or 84 mg) through weeks 9 to 32, all in addition to an ongoing OAD.

^b^Quetiapine XR was dosed once daily, starting at 50 mg and titrated up to ≥150 mg/day by the end of week 2 and then flexibly dosed (150 to 300 mg/day) from weeks 3 to 32, all in addition to ongoing treatment with an OAD.

**Figure S2. CONSORT Diagram**

**
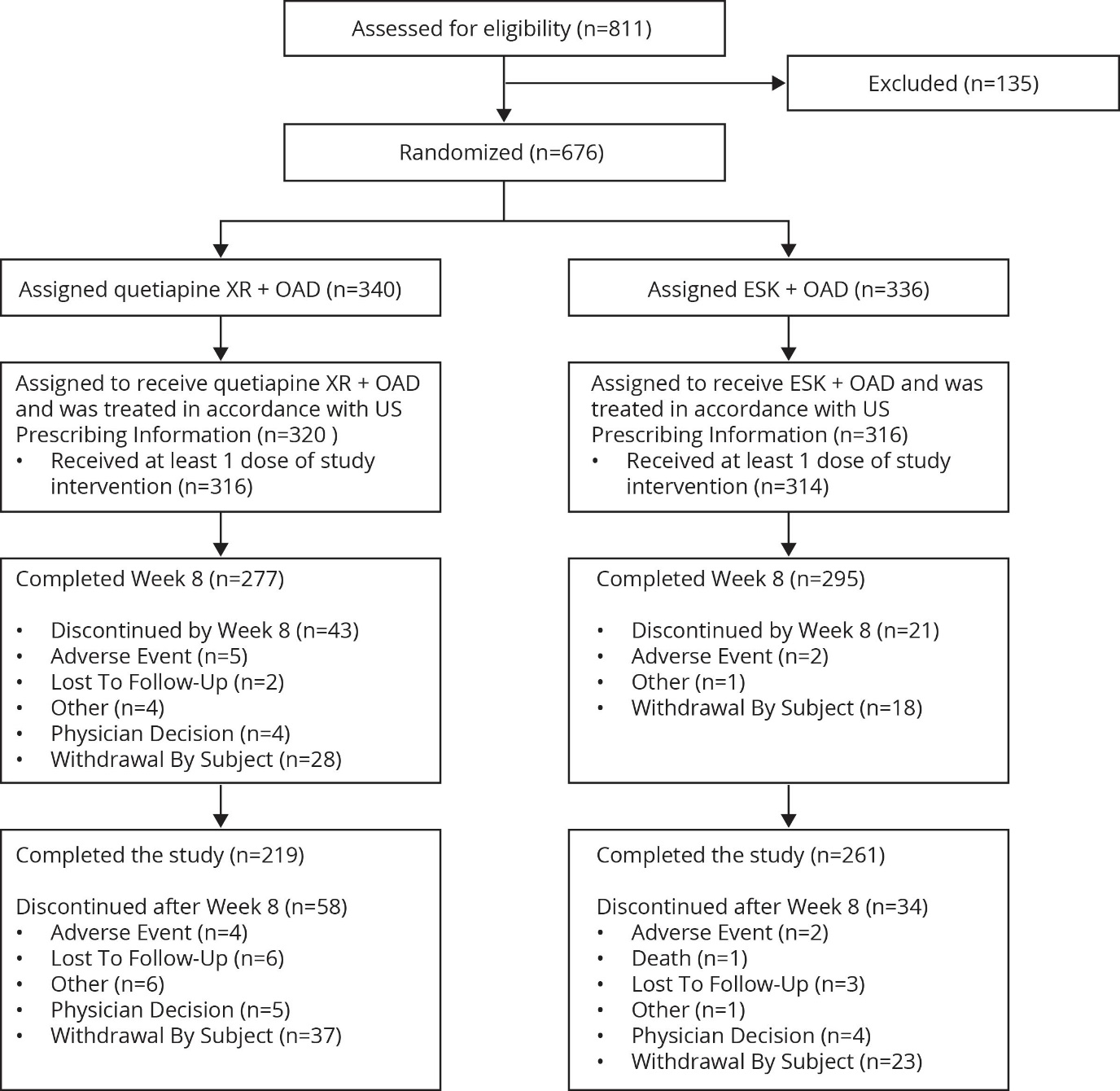
**

Abbreviations: ESK, esketamine nasal spray; OAD, oral antidepressant; XR, extended-release.

**Figure S3. LS Mean Change in MADRS Total Score Over Time**


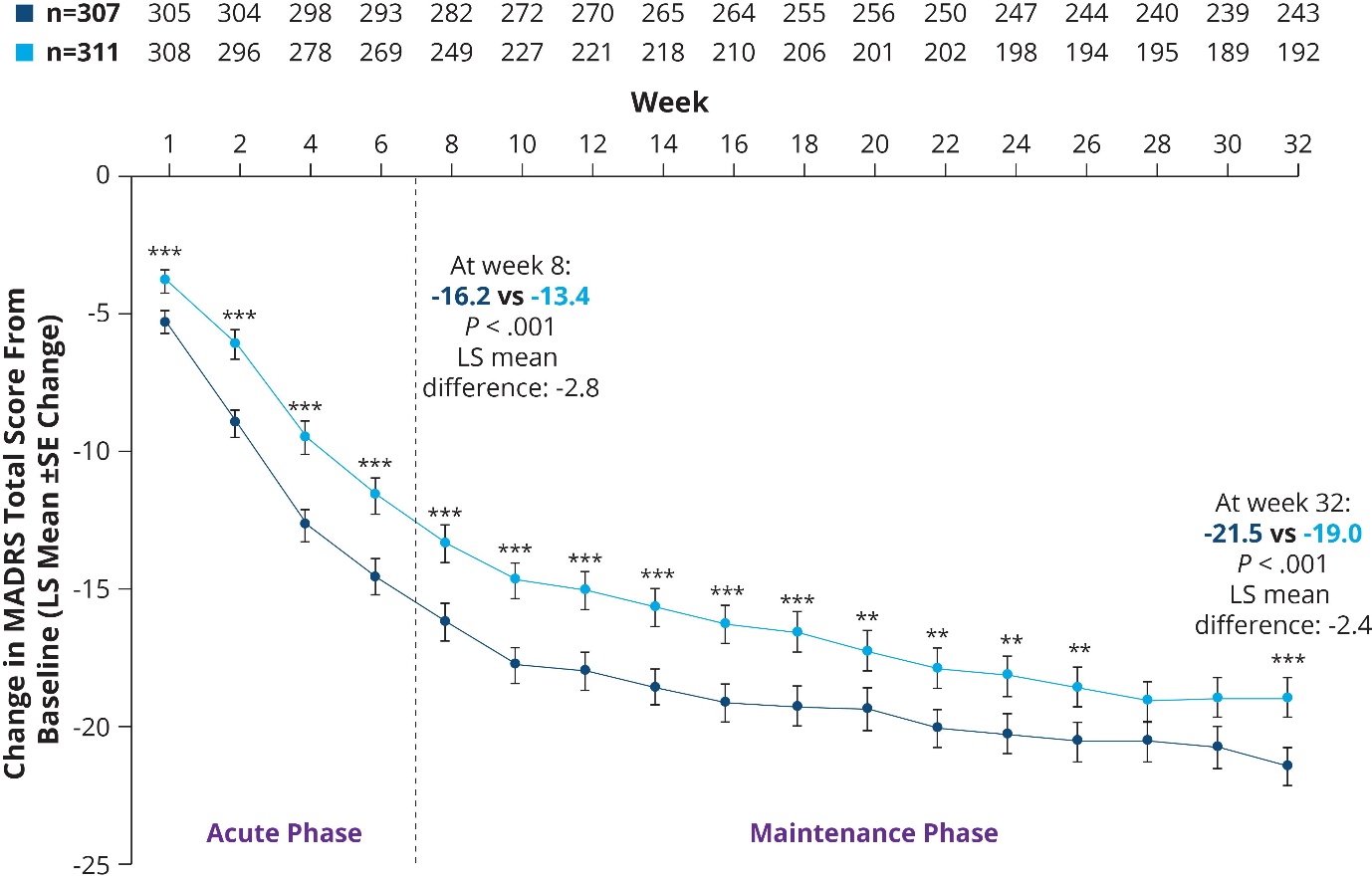


Abbreviations: ESK, esketamine nasal spray; LS, least squares; MADRS, Montgomery-Åsberg Depression Rating Scale; MMRM, mixed models for repeated measures; OAD, oral antidepressant; OC, observed cases; SE, standard error; XR, extended-release.
The full analysis set includes all randomly assigned patients. Error bars depict SEs. LS means and SE were based on MMRM (based on OC), with treatment, number of treatment failures, time, time by treatment, and baseline value as covariates. Correlations within patients were modeled with an unstructured covariance structure.
***P* < .01; ****P* < .001.

**Figure S4. Proportion of Patients Achieving the Primary Endpoint per Sensitivity Analysis**


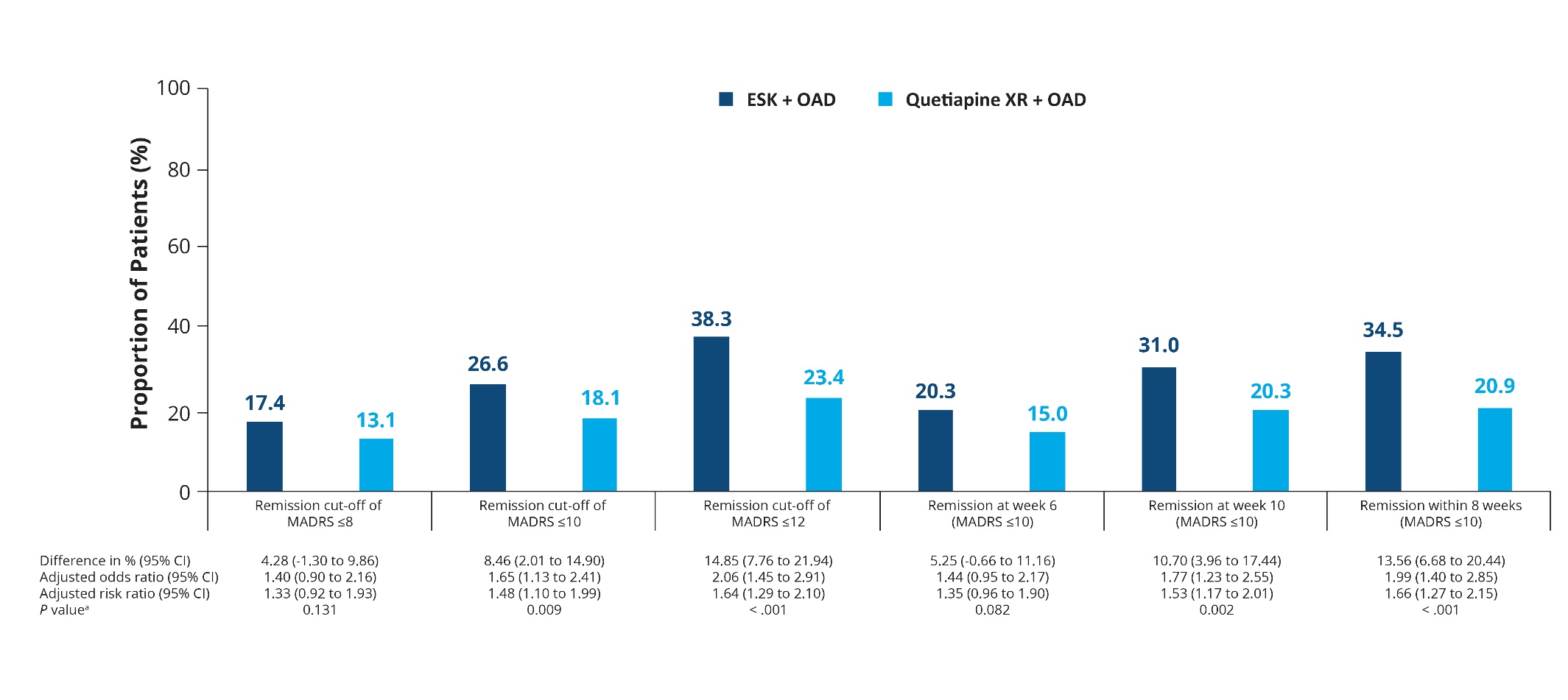


Abbreviations: CI, confidence interval; ESK, esketamine nasal spray; MADRS, Montgomery‑Åsberg Depression Rating Scale; OAD, oral antidepressant; XR, extended-release.
Percentages are based on the number of subjects in the full analysis set.
The primary endpoint was achieving remission (MADRS total score ≤10) at week 8.  ^a^*P* value for Cochran-Mantel-Haenszel test adjusting for total number of treatment failures (2; ≥3).

**Figure S5. Proportion of Patients Achieving the Key Secondary Endpoint per Sensitivity Analysis**


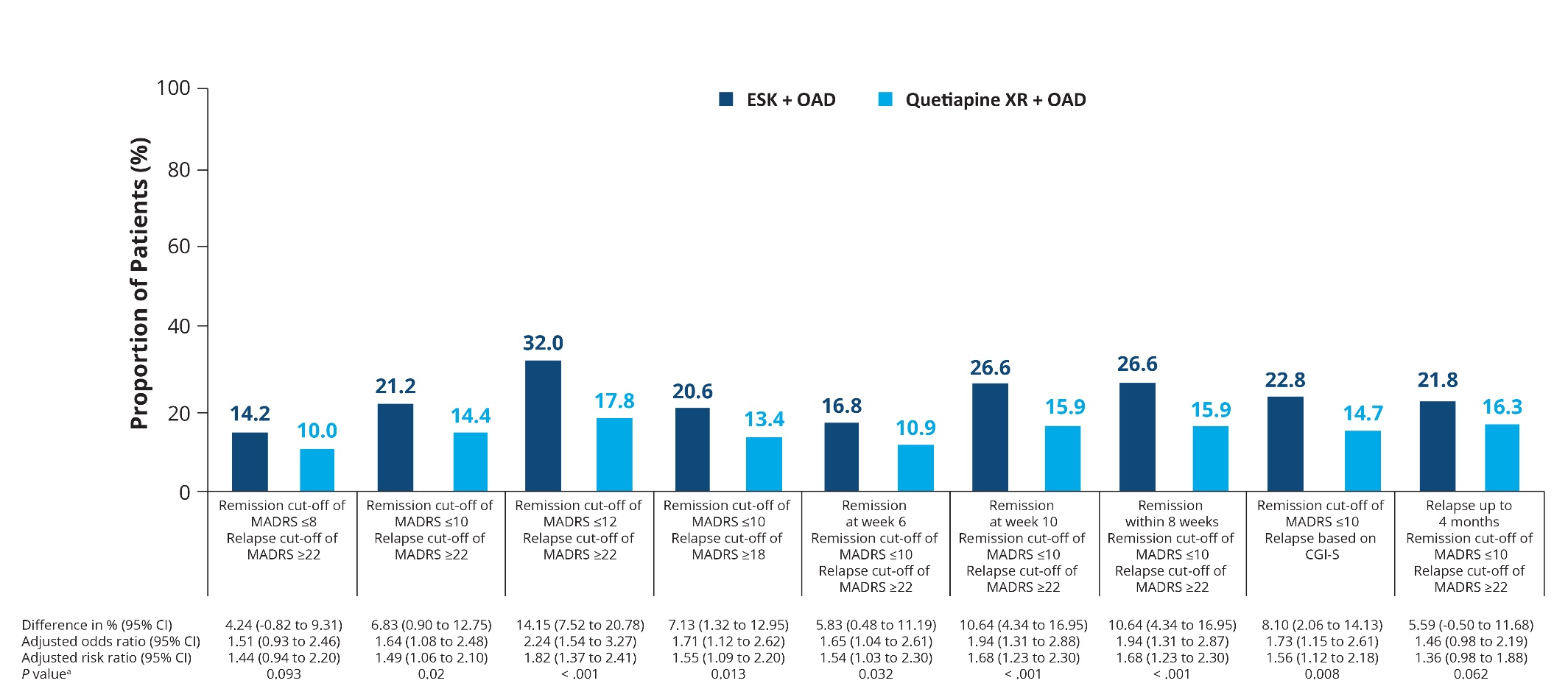


Abbreviations: CGI‑S, Clinical Global Impression‑Severity scale; CI, confidence interval; ESK, esketamine nasal spray; MADRS, Montgomery‑Åsberg Depression Rating Scale; OAD, oral antidepressant; XR, extended release
Percentages are based on the number of subjects in the full analysis set.
The key secondary endpoint was remaining relapse‑free though week 32 after achieving remission at week 8.
For relapse based on CGI-S, relapse was defined as any of (a) CGI-S total score of ≥5, (b) hospitalization for worsening of depression, or (c) suicide attempt, completed suicide, or other clinically relevant event not associated with hospitalization.  ^a^*P* value for Cochran-Mantel-Haenszel test adjusting for total number of treatment failures (2; ≥3).

**Figure S6. Patients in Remission Based on MADRS Total Scores ≤8, ≤10, and ≤12 Over Time (LOCF)**


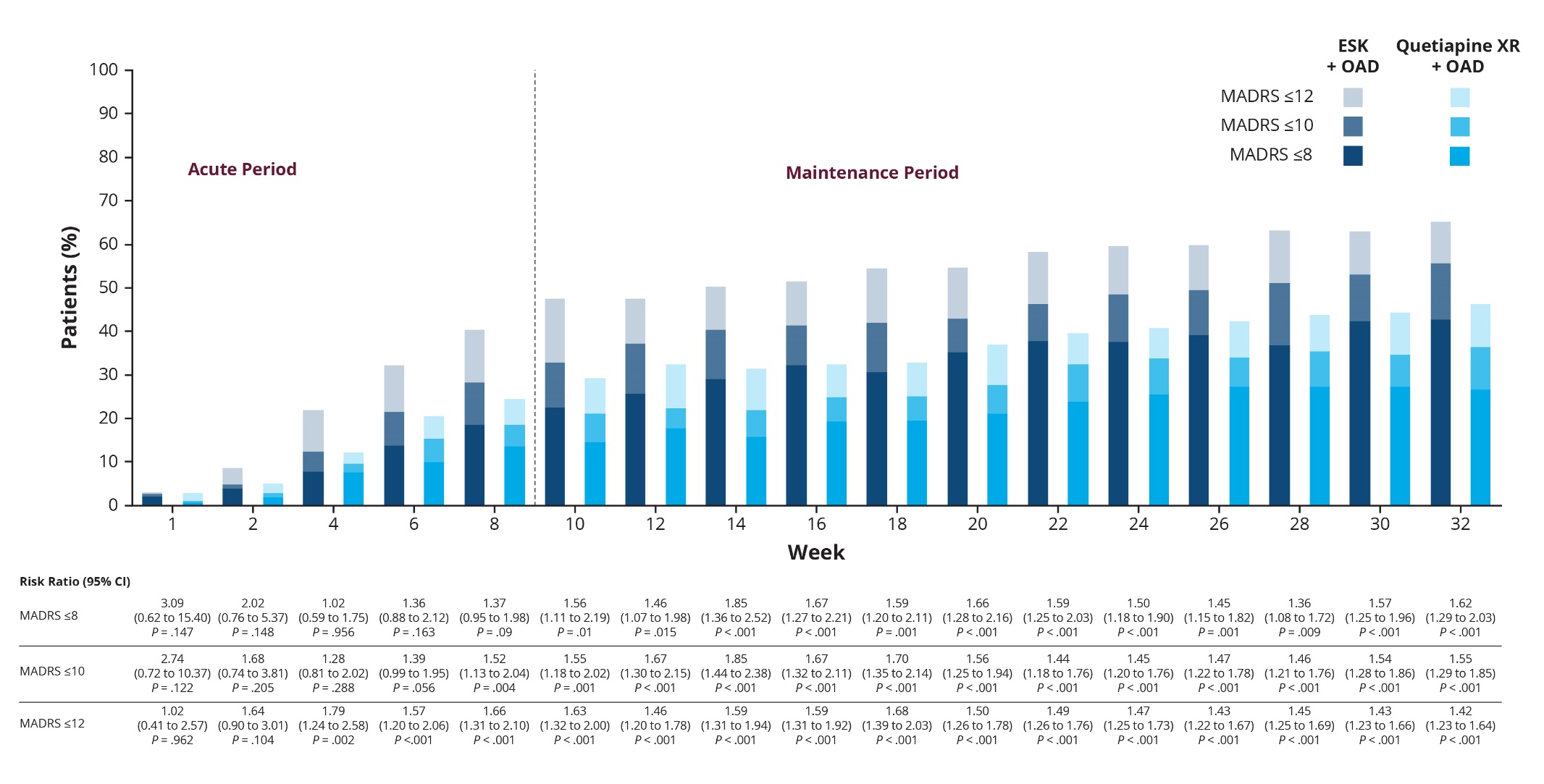


Abbreviations: CI, confidence interval; ESK, esketamine nasal spray; LOCF, last observation carried forward; MADRS, Montgomery‑Åsberg Depression Rating Scale; OAD, oral antidepressant; XR, extended-release.
Percentages are based on the number of subjects at each timepoint for LOCF. Remission is defined as a MADRS total score ≤8, ≤10, or ≤12. *P* value for Cochran-Mantel-Haenszel test adjusting for total number of treatment failures (2; ≥3).
